# An Ecological Model of Care for Older Adults With Cognitive Impairment

**DOI:** 10.1093/geroni/igaa057.503

**Published:** 2020-12-16

**Authors:** Elizabeth Rhodus, Graham Rowles, Shani Bardach, Elizabeth Hunter, Gregory Jicha

**Affiliations:** 1 University of Kentucky, Richmond, Kentucky, United States; 2 University of Kentucky, Lexington, Kentucky, United States; 3 University of Kentucky, University of Kentucky, United States

## Abstract

Living with dementia causes increasing dependence on the surrounding physical and social environment. There is limited information on environmental interactions of persons with cognitive impairment. Based on participant observation and repeated interviews with both members of nine dyads (primary care partner and person with cognitive impairment) in situ in their homes, a theoretical model depicting environmental interaction was developed. The model illustrates parallel and interwoven environmental experiences of each member of the dyad as they negotiate progressive cognitive impairment. Evolution of the dyad is situated within nested layers of the physical and contextual environment including physical structures, social norms, and political environments. Experiential elements for each member of the dyad are described. Elements include cognitive status, trial and error associated with care provision, adverse behavior linked with onset of caregiver burden, onset of a significant event leading to altered living situations, and maximum dependence on environmental factors prior to end of life. Evidence collected suggests that both persons of the dyad become increasingly susceptible to environmental influences with progression of the disorder. Implication of these findings offer a theoretical framework describing dyadic experiences of environmental interactions when living with dementia. This theoretical model provides a basis for clinical and social intervention to enhance the well-being of both members of the dyad. Interventions associated with environmental interactions may slow socially discordant behavioral manifestations associated with dementia and significantly improve quality of life for both members of the dyad.

